# Patients’ Perceptions of Using a Digital Previsit Tool in Outpatient Settings (Part 2): Qualitative Study

**DOI:** 10.2196/73477

**Published:** 2025-10-06

**Authors:** Petra Pohl, Emma K Kjörk

**Affiliations:** 1 Department of Health and Rehabilitation Sahlgrenska Academy, Institute of Neuroscience and Physiology University of Gothenburg Gothenburg Sweden; 2 Department of Clinical Neuroscience Institute of Neuroscience and Physiology, Sahlgrenska Academy University of Gothenburg Gothenburg Sweden; 3 Centre for Person-Centred Care (GPCC) University of Gothenburg Gothenburg Sweden; 4 Sahlgrenska University Hospital Gothenburg Sweden

**Keywords:** stroke rehabilitation, post-stroke checklist, person-centered care, follow-up, eHealth, health literacy, patient engagement

## Abstract

**Background:**

Patients with long-term conditions, such as stroke, require regular follow-up visits to health care professionals to identify changes in symptoms. The digital previsit tool Strokehälsa (Strokehealth) has been designed to encourage individuals with stroke to reflect on stroke-related health concerns before a follow-up visit, thereby potentially enhancing their engagement during the visit. Strokehealth has previously been evaluated using a patient satisfaction survey (part 1), but there remains a need to further explore patients’ perceptions and needs to optimize its functionality before broader implementation.

**Objective:**

The overall aim was to attain deeper insights into patients’ views and experiences of using the digital previsit tool Strokehealth before a follow-up visit. A secondary aim was to identify potential improvements to the tool based on these insights.

**Methods:**

For this qualitative study, patients who had used Strokehealth version 1.0 before a follow-up visit were recruited through the previous survey between November 2020 and June 2021. Individual semistructured interviews were conducted, and data were analyzed using reflexive thematic analysis. Subsequent workshops were held with people with firsthand experiences of stroke, other stakeholders (including health professionals and researchers), and a web consultant to finalize decisions regarding adjustments to be implemented in Strokehealth version 2.0.

**Results:**

Interviews were conducted with 33 participants (23 men and 10 women), with a median age of 67 (IQR 55-76) years. Analysis of the data regarding participants’ experiences of using Strokehealth revealed three overarching themes: (1) a supporting tool for preparing dialogue and identifying needs, (2) how Strokehealth is introduced and communicated affects perceived usability, and (3) the wording and structure of Strokehealth influences the response process. The findings captured various aspects of receiving and using the digital previsit tool, highlighting its simplicity and purpose. Overall, Strokehealth was well received and contributed to a sense of being well cared for. Participants generally not only found Strokehealth easy to use but also shared suggestions on how to better address stroke-related issues, such as mental fatigue or pain. Examples of changes that have been implemented in Strokehealth version 2.0, based on participant feedback, include improved explanatory texts and expanded opportunities for free text.

**Conclusions:**

The findings indicate that the freely available digital previsit tool Strokehealth was generally well received by patients with stroke who were scheduled for follow-up visits in outpatient settings.

**Trial Registration:**

Researchweb 275135; https://www.researchweb.org/is/vgr/project/275135

## Introduction

Health care systems are increasingly adopting digital support to address future challenges and to transition toward more person-centered care [1]. This shift has prompted a significant increase in the development and use of digital services and tools for self-care among community-dwelling people [2]. Advancements in medical care and improvements in health care services are leading to rising numbers of individuals living with conditions that require long-term care, such as stroke [3]. In patients with such conditions, a lack of adequate self-care or health care interventions can lead to serious consequences [3]. Continuous access to health care services is essential to ensure optimal self-management, psychological and emotional support [4,5], and treatment for patients, regardless of their condition [6]. The integration of digital technology can potentially enhance easy access to health care, patient engagement, and self-management [2,7]. However, individuals managing long-term conditions commonly show low eHealth literacy, potentially resulting in their exclusion from digital health resources [8]. Therefore, it is imperative that digital tools be tailored specifically for individuals with diverse health conditions and be designed with enhanced usability and accessibility.

Regular follow-up visits enable caregivers to identify potential health problems, detect symptom changes or worsening at an early stage, and take preventive actions to minimize their impact [9]. Follow-up visits also serve as opportunities to provide patients with support, education, and counseling regarding their condition, treatment options, and symptom management. This helps patients better understand their condition and empowers them to take a more active role in their health care. During follow-up visits, meaningful conversations can be facilitated using tools such as checklists or dialogue aids, for example, the Post Stroke Checklist [10]. However, patients have emphasized the importance of advance preparation to enhance engagement during discussions with health care professionals [11]. Such preparation can be achieved through previsit tools [12,13], which encourage patients to reflect in advance, and thereby make interactions with health care providers more efficient. Accordingly, a previsit tool for individuals with stroke—Strokehälsa (“Strokehealth”), based on the Post Stroke Checklist—was developed in a participatory co-design process with a patient partner, stakeholders, and a service designer [14]. The aim was to create a tool that would enable people—including those with various disabilities—to take an active part in their decision-making process by providing them with targeted questions and time to reflect on stroke-related concerns before their scheduled visit to an outpatient clinic specialized in stroke care [14]. Other previsit tools exist but most are designed to collect physical health data, such as blood glucose levels, or are highly comprehensive (eg, the stroke-related “Rehabkompassen” [15]), and require substantial time and effort from the user. In contrast, Strokehealth was designed to support reflection and communication, rather than extensive data collection.

In short, the content of Strokehealth and the procedure for its use in health care can be described as follows. First, the patient is introduced to Strokehealth version 1.0 through a brief introductory text before answering 14 questions (Multimedia Appendix 1) covering various health-related domains. Each question includes a short explanatory text to clarify and provide examples. To indicate any health concerns, the response choices are “yes,” “no,” or “choose not to answer.” Upon completing Strokehealth, patients have the opportunity to provide additional open-ended comments or information before their scheduled visit. In addition, patients are offered further advisory information about stroke and self-management strategies, which is accessible via a clickable link. In clinical practice, health care providers send Strokehealth to patients via the Swedish national patient portal, prompting them by email or text message to log in and complete the tool. The health care provider—usually a stroke-specialized nurse—then has the option to review the patient’s responses in advance and, if necessary, consult other professionals—for example, a physician or an occupational therapist—prior to the appointment, in order to proactively address the patient’s concerns, although this step is optional.

In the development and evaluation of complex interventions, contextual factors—including organizational and local conditions—play a decisive role [16]. Therefore, Strokehealth was evaluated in a real-world setting involving patients who were discharged from a stroke unit and scheduled for follow-up visits. A previous study (part 1) was conducted using a web-based patient satisfaction survey, revealing that Strokehealth was perceived to provide a structured framework that facilitated the consideration of important issues in conjunction with health care visits [17]. A wide range of health problems was identified, and 56 out of 58 (96.6%) participants expressed confidence that Strokehealth effectively captured their health-related concerns. Analysis of free-text responses revealed that Strokehealth was viewed as a valuable tool for structured follow-up and was described as enabling increased patient engagement. Participants also noted that Strokehealth provided valuable information, offering enhanced insights into their condition and the available support. Suggestions for improvement included extended response options, the opportunity to develop one’s answers, and clearer explanatory texts [17]. Sustainable development and implementation of the previsit tool require evaluating feasibility from different perspectives, especially from the patients’ perspective as end users. Thus, it was considered necessary to conduct individual interviews to explore the participants’ survey responses in depth and to allow them to elaborate on their suggestions for improvements. The present interview study is an important step in the development and evaluation of Strokehealth before its implementation in health care.

In this study, we aimed to gain deeper insights into patients’ experiences with and views on using the Strokehealth digital previsit tool before follow-up visits. A secondary aim was to identify further ways to improve the previsit tool based on these findings.

## Methods

### Study Design

A qualitative study was conducted [18] as part of a larger research project aimed at developing and evaluating the Strokehealth previsit tool, using quantitative and qualitative methods and following the Medical Research Council framework [16]. The research paradigm guiding this study, as part of the larger project, is inspired by the principles of realistic evaluation based on scientific realism, in which various data sources are used in a pragmatic and flexible manner to explore “what works, for whom, under what circumstances?” [19]. This study involved the interpretative analysis of semistructured interviews to attain insights regarding how different contexts and mechanisms influence outcomes and to inform changes to the previsit tool. In this study, the COREQ (Consolidated Criteria for Reporting Qualitative Research) checklist was used to ensure explicit and comprehensive reporting (Multimedia Appendix 2) [20].

### Ethical Considerations

Ethical approval was obtained from the Swedish Ethical Review Authority (556-17 and 2020-03324). After logging into the patient portal, patients were presented with information regarding the research project and asked whether they would consent to participate. Only those who provided informed consent were able to proceed with the survey (part 1) [17]. The introductory information made it clear that participation was voluntary and would not impact their medical treatment. No incentives were offered for participation. At the end of the survey, participants were asked a follow-up question about participating in an interview. All transcripts were anonymized before analysis and treated confidentially because they contained health-related information. Only the research team had access to the interview recordings, transcripts, and consent forms. No prior relationship was established between the researchers and participants before the interviews.

### Setting and Participants

**Figure 1:**
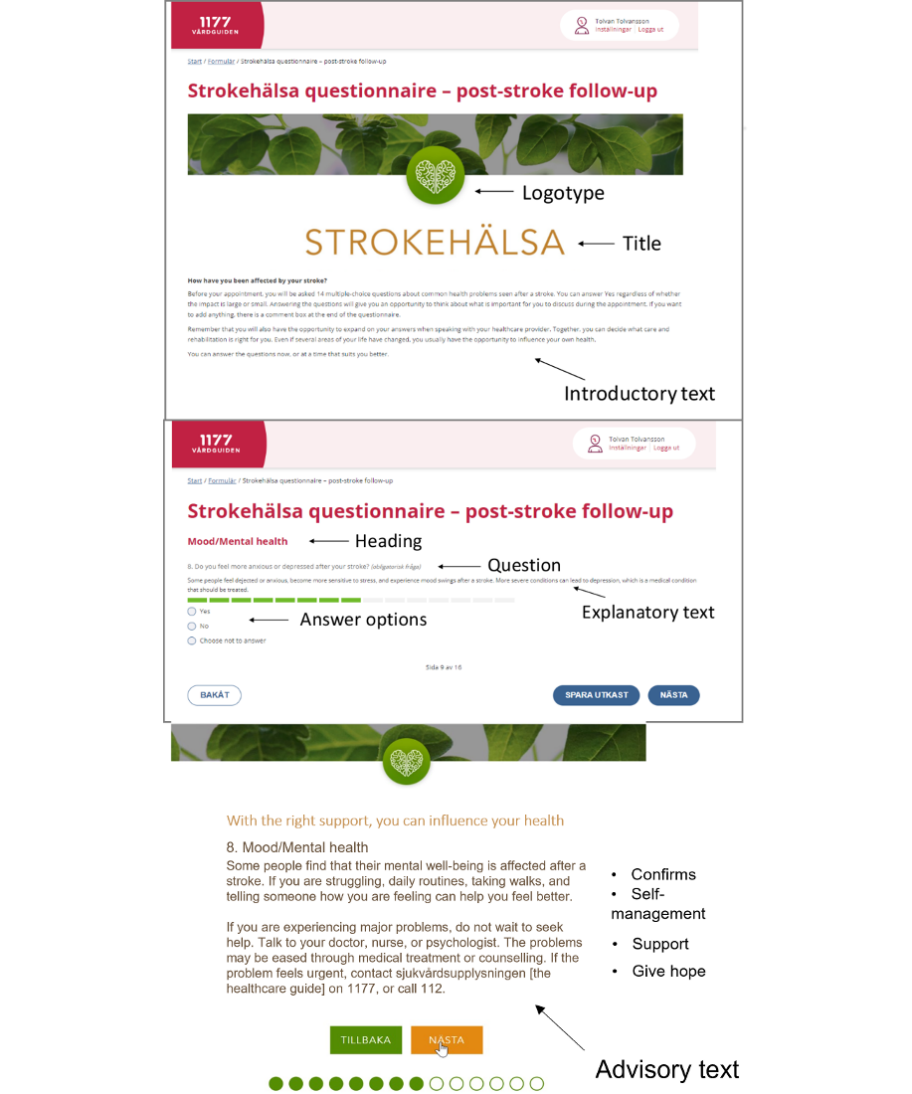
A screenshot of the elements in the previsit tool Strokehälsa (English version; reproduced from Kjörk et al [14], which is published under Creative Commons Attribution 4.0 International License [21].

This study was conducted in the Västra Götaland region of Sweden. The study participants were community-dwelling individuals who had experienced a stroke, had recently used Strokehealth (Figure 1), completed a subsequent patient satisfaction survey, and volunteered to share their more in-depth experiences regarding the tool. Initial participant recruitment was performed consecutively between November 2020 and June 2021 from 3 hospitals in the region. The inclusion criteria were admission to a stroke unit, being scheduled for a follow-up visit to a stroke team nurse after hospital discharge, and having a personal account on the Swedish national patient portal. No exclusion criteria were set. Strokehealth was sent approximately 3 weeks to 3 months after discharge from the hospital, depending on the standard routines of the outpatient clinic.

A web-based survey was sent to all patients who had used the tool (n=80). The survey was completed by 73% (58/80; 39 men; age: median 67, IQR 56-75 years). The survey included information about its purpose: to gain a better understanding of their experiences using the previsit tool Strokehealth in preparation for their follow-up visit, with a focus on its usability and functionality. At the end of the survey, respondents were asked whether they consented to future contact from the research team regarding a more in-depth interview. It was emphasized that the interviews would contribute to improving follow-up care supported by the tool. A total of 36 patients indicated their interest in further collaboration and were contacted via telephone by the last author (EKK), of whom 33 agreed to participate in individual interviews.

### Data Collection

Data were collected using a semistructured interview guide, including questions about experiences and aspects of using Strokehealth (Multimedia Appendix 3). The interview guide was pilot-tested, and no adjustments were deemed necessary; therefore, the pilot interview was included in the present analysis. All interviews were conducted in Swedish over the telephone at prearranged dates and times. At the time of the interviews, only a few participants had completed their follow-up visits. No relationship had been established between the interviewer and the participants before the interviews. The participants’ next of kin were allowed to be present and to participate when appropriate. During 2 interviews, a spouse was present for support.

All interviews were audio recorded. The study aims were repeated, and the participants orally confirmed their consent. To ensure high credibility, all interviews began with the same introduction, emphasizing the importance of the participants’ experiences and viewpoints and that there were no right or wrong answers. Prompts such as “Could you elaborate on your thoughts?” were used. The duration of the interviews ranged from 8 to 40 minutes (median 24 minutes, IQR 21-28 minutes). All interviews were transcribed verbatim by a person who was not part of the study, using MS Word. The transcripts comprised 289 pages of single-spaced text. No member checks were conducted, and participants were not asked to provide feedback on the results.

### Data Analysis

A reflexive thematic analysis [22] was conducted, aligning with an interpretivist paradigm that emphasizes understanding individuals’ subjective experiences [18]. Reflexive thematic analysis was deemed an appropriate analytical approach based on the study’s focus on the unique experiences of the patients who had used Strokehealth. Furthermore, reflexive thematic analysis entails a flexible process that facilitates the identification, analysis, and reporting of patterns of meaning within interview data [22]. In reflexive thematic analysis, both coding and theme development are flexible processes that evolve throughout the analysis. An inductive approach was used, that is, the analysis was data driven and based on the participants’ subjective experiences. Analysis was performed by 2 authors (EKK and PP) using the NVivo software (version 12; Lumivero).

The analysis was performed using the 6-stage framework described by Braun and Clarke (Textbox 1) [22]. Throughout the analysis process, conscious reflections were made, and reflexive thematic analysis facilitated the authors’ reflective engagement.

**The 6-stage framework of reflexive thematic analysis.**

Stage 1. Both authors independently read and reread all transcripts to become familiar with the data.

Stage 2. Important features related to the research question were identified within the dataset and coded according to their content.

Stage 3. The codes were examined to identify significant patterns of meaning and clustered into potential themes and subthemes.

Stage 4. Themes and subthemes were reviewed against the dataset, and redefined in an iterative process, with continuous back-and-forth between the main texts and the codes.

Stage 5. The themes were analyzed in detail to refine their scope and focus, and informative names were assigned.

Stage 6. Analytical narrative and data extracts were contextualized in relation to existing literature, and the manuscript was produced.

After the completion of the analysis, some of the suggested changes that emerged from the interviews were implemented in Strokehealth, resulting in version 2.0. This process included consultation with members of the advisory board who were involved in the previous study [14]. In addition, a linguist reviewed the text to improve its clarity and structure. Finally, the proposed amendments to Strokehealth were discussed in formal decision-making workshops with the project’s research team in collaboration with the patient partner. After the final decision workshop, adjustments to Strokehealth in the patient portal were made through close collaboration between EKK and a web designer. These workshops guided the final decisions about the design of Strokehealth and addressed issues such as clarifying the language by adding or removing text and answer options.

### Research Team and Reflexivity

The interviews were conducted by EKK, a female who holds a PhD degree in occupational therapy and is a senior lecturer. She is an established researcher with previous experience in qualitative research and is well published in the fields of stroke rehabilitation and eHealth. Transcripts were cross-checked by PP, a female who holds a PhD degree in physical therapy. She is an assistant professor with previous experience in qualitative research and is well published in the fields of neurological rehabilitation and eHealth.

## Results

### Participants

The 33 study participants included 23 men and 10 women, with a median age of 67 (range 43-87) years (Table 1). Among these participants, 28 (85%) reported using the web multiple times per day, 3 (9%) reported using it weekly, and 2 (6%) reported using it once per year. Four participants (12%) received assistance in completing Strokehealth.

**Table 1:** Participant characteristics.

Characteristics			Participants in interviews (n=33)
**Age (years)**			
	All patients, median (minimum-maximum)	67 (43-87)	
**Sex, n (%)**			
	Male	23 (70)	
	Female	10 (30)	
**Education (highest level), n (%)**			
	Mandatory	10 (30)	
	High school	8 (24)	
	University	14 (42)	
	Other	1 (3)	
**Source of income at inclusion, n (%)**			
	Work	13 (39)	
	Sick leave	2 (6)	
	Retirement	17 (52)	
	Other	1 (3)	
**Prestroke living conditions, n (%)^a^**			
	Without assisted care in own home	32 (100)	
	Living alone	5 (15)	
	Independent in activities of daily living	30 (94)	
**Stroke characteristics (onset)**			
	Cerebral infarct, n (%)	31 (94)	
	Intracerebral hemorrhage, n (%)	2 (6)	
	Previous stroke, n (%)	5 (15)	
	Stroke severity, NIHSS^b^, median (minimum-maximum)	1 (0-7)	
**Stroke-related outcomes**			
	Length of hospital stay in days median (minimum-maximum)	5.5 (1-35)	
	Discharged to own home, n (%)	33 (100)	

^a^Missing data: without assisted care in own home (n=1), independent (n=1).

^b^NIHSS: National Institutes of Health Stroke Scale; measured ≤24 hours of admission, normal values 0-42. Values are presented as numbers and valid percentages unless stated otherwise.

### Themes Created Based on the Individual Interviews

**Figure 2:**
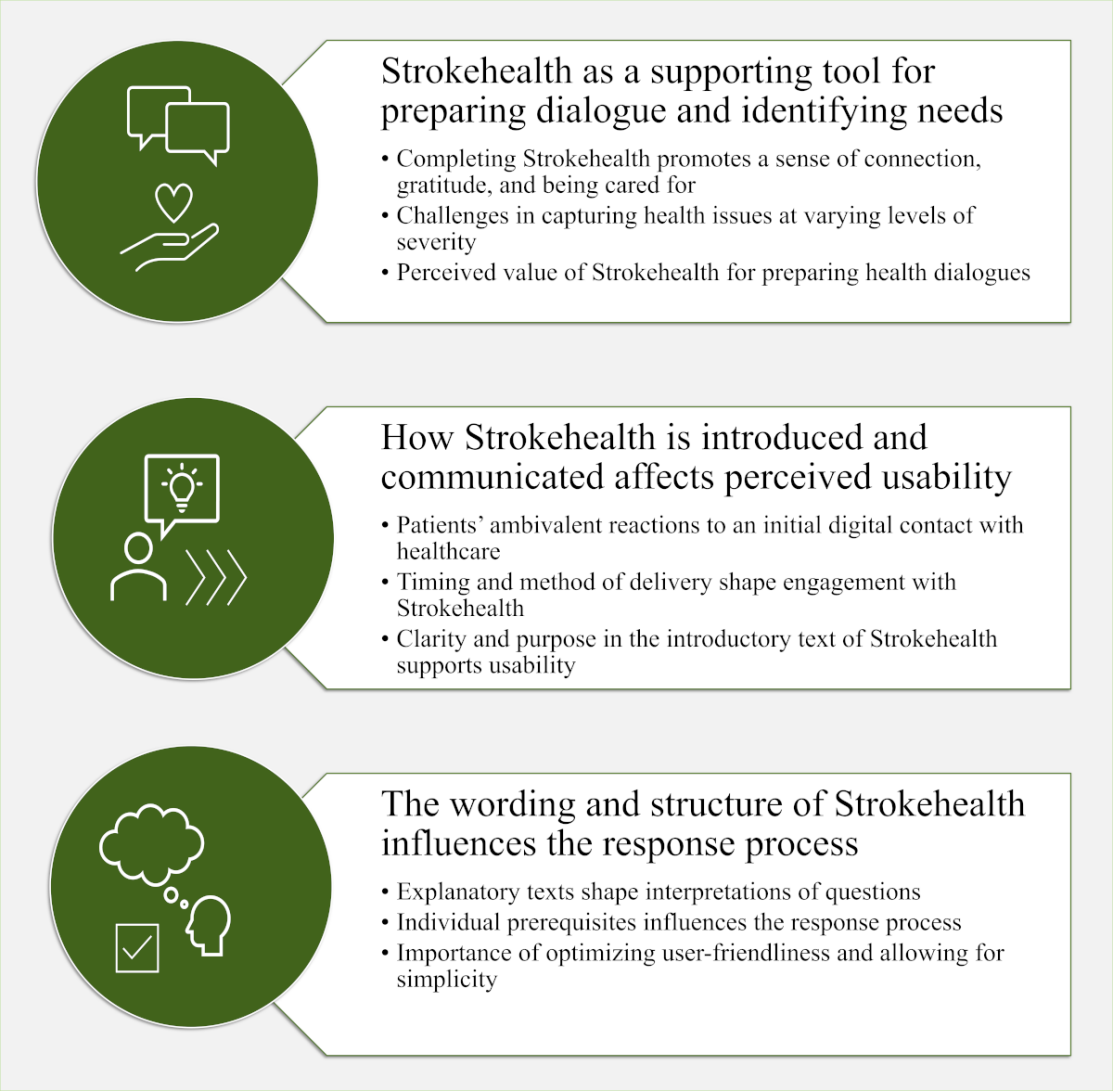
Themes and subthemes identified from interviews regarding the digital previsit tool Strokehealth.

The analysis resulted in the identification of 3 main themes, reflecting the participants’ overall experiences with receiving and using the previsit tool before their planned follow-up visit to the nurse. The three main themes were as follows: (1) Strokehealth as a supporting tool for preparing dialogue and identifying needs, (2) how Strokehealth is introduced and communicated affects perceived usability, and (3) the wording and structure of Strokehealth influences the response process (Figure 2).

#### Strokehealth as a Supporting Tool for Preparing Dialogue and Identifying Needs

This main theme represents aspects of how effectively Strokehealth captured the participants’ health issues and how it was perceived in preparing patients for dialogue with health care professionals as well as family members.

##### Completing Strokehealth Promotes a Sense of Connection, Gratitude, and Being Cared for

The use of Strokehealth as preparation for a visit was perceived to contribute to the sense of being well cared for by health care. Some participants felt reassured by encountering familiar information within the tool, as this validated their existing knowledge and aligned with previous information received from health care providers. This recognition created a sense of connection and reassurance that they were not alone, and that experiencing such issues after a stroke was both expected and normal. Others expressed that the information provided within Strokehealth helped to fill knowledge gaps, supplemented their existing understanding, and brought a sense of hopefulness.

Participant 2: It’s really nice, what I’m looking at right now [advisory text]; it looks really good. It’s short, concise, pleasant, and gives hope [laughs]. No, but I think so. I don’t really have anything to object to. You can’t do more than this, really. It looks nice, good, and informative. [Male, 61 years old]

Completing Strokehealth also evoked feelings of gratitude for being taken seriously by the health care system and for receiving good care. It provided participants with an opportunity to actively reflect on what it meant to have survived a stroke. They felt grateful for not being more severely affected by the stroke and for the confirmation that it could have been much worse, as they read about different problem areas—one question after another.

##### Challenges in Capturing Health Issues at Varying Levels of Severity

Most participants felt that the questions in Strokehealth were relevant and adequately addressed their health issues, including mental fatigue. However, individuals experiencing very mild symptoms perceived that the questions or the given examples did not fully capture their own experience. This prompted reflections on whether Strokehealth might be better suited for individuals with more severe strokes. One participant experiencing mild aphasia found that none of the questions adequately addressed this specific problem.

Participant 15: It also depends on what level I’m answering from. If I’m answering from an everyday perspective—can I go to the store, talk to my family and friends, participate in Teams meetings, or write messages, emails, or texts—then I have no problems. But if I want to do it at a higher level, workwise, maybe speak another language, and so on, then that’s a completely different level, and perhaps a completely different answer to the question. [Male, 43 years old]

Another participant mentioned that none of the questions captured the challenges of everyday tasks that were previously effortless, such as emptying the dishwasher. Now, she had to consider each step while maintaining balance, which made the task exhausting. Participants suggested that more generous open-response areas would allow for detailed explanations of such issues. Participants also found that the questions in Strokehealth were insufficient to capture psychological factors, such as worrying thoughts about the future. They noted that the question related to this topic did not precisely reflect what participants wanted to express.

##### Perceived Value of Strokehealth for Preparing Health Dialogues

Participants found that completing Strokehealth not only helped them prepare for their follow-up visit but also provided them with a valuable opportunity to reflect on their situation and thoroughly consider their individual needs. They also felt that the tool helped make them aware of certain issues that might not have otherwise been addressed, such as their relationship with loved ones. Some participants even expressed that Strokehealth was valuable for explaining their problems to family members and highlighting common poststroke issues. They suggested that a previsit tool like Strokehealth could be beneficial for structuring and optimizing any health care visit that is often time-limited.

Participant 4: It makes things easier; it becomes like a preparation beforehand. I guess that’s the case with all meetings—the more prepared you are, the better it is. If you know what it will be about and can reflect on it beforehand, it’s always beneficial. So, I think that’s a good thing. [Male, 58 years old]

Participants also reflected on the potential value to the staff if patients filled out Strokehealth, speculating that it might make the meeting more efficient. However, not everyone believed that Strokehealth was necessary; some felt confident about directly articulating their concerns and believed that the nurse was already informed about their problems.

Furthermore, Strokehealth also introduced new vocabulary that inspired them to seek further information in advance—for example, through search engines such as Google—to learn more about their condition and possible interventions. Consequently, this process of independently seeking information further enhanced their preparedness for the upcoming health care encounter.

#### How Strokehealth Is Introduced and Communicated Affects Perceived Usability

This main theme emphasizes that the way Strokehealth is introduced and communicated to patients plays a crucial role in how the tool is perceived and used. The analysis underscores the significance of offering patients various options for appointment preparation and enabling them to make active choices.

##### Patients’ Ambivalent Reactions to an Initial Digital Contact With Health Care

Participants described different reactions upon receiving the digital notification, that is, a text message on their smartphone indicating that there is a form to complete (Strokehealth). Many participants who were already familiar with the patient portal became curious and felt confident that the short text message was sent from a trustworthy source. However, the meaning of the text message was not always clear to participants who had never previously received a notification from the patient portal. Some participants felt a sense of suspicion, thinking that an authority wanted to poke around in their private life or had concerns about the risk of fraud, as often reported in the media. In some cases, the sender (the nurse from the stroke team) would follow up with a telephone call. These verbal reminders increased patients’ motivation to log in and complete Strokehealth. However, some participants mentioned that they would have preferred the initial contact after their stroke to be with a real person rather than an anonymous text message prompting them to answer questions using a digital tool, while others were fully content with the “digital-first” approach.

Participant 19: Well, I’m a bit critical and thinking like this—I was at the stroke unit about 14 days ago, and this is the first contact, about ten days later, that I receive from the healthcare side, and I find that a bit contradictory. On the one hand, maybe one would have wanted to talk to a person and not fill out a form because, after all, it’s a transformative experience to have a stroke. [Male, 63 years old]

Participant 30: I felt that it’s really good. I think all follow-up feels reassuring. It’s very intense when it happens, and then the healthcare system kicks in, and suddenly, you’re back home. I think all follow-up is good. And digital tools are totally fine, especially in combination with actually talking to someone, like in this case [Strokehealth combined with a dialogue with a nurse]. So, no, I’m in favor. [Male, 45 years old]

Several participants expressed that they would have preferred to be informed about Strokehealth during their hospital stay rather than only through a digital notification. However, they also acknowledged that this information might have been overlooked due to the many other concerns during their hospitalization.

##### Timing and Method of Delivery Shape Engagement With Strokehealth

Participants had various thoughts on how to personalize the process of when and how to complete Strokehealth. For instance, they reflected on the time span between hospital discharge and receipt of Strokehealth. It was generally considered that one needed some time to settle back home after discharge before the follow-up visit was scheduled and Strokehealth was delivered.

Participants also reflected on the timing of receiving the text message with a prompt to open the tool. Most considered the forenoon to be appropriate because they still had some of their often-limited energy left. If the message was sent during the night, the signal was likely to wake the recipient. Participants also emphasized that they wanted the flexibility to choose the most convenient time of day to engage with the tool. Approximately 1 week of preparation time was generally considered sufficient to allow participants to select the best time of day to engage with Strokehealth.

Participant 11: And then you could complete it at your own pace as well, you didn’t have to rush through the questions, so it’s quite nice, so yeah, I think it was a good thing, a really good thing. [Female, 53 years old]

Participants generally appreciated that they could log in and complete Strokehealth using a device of their own choice. The text was considered sufficiently visible on any device—including computers, tablets, and smartphones. However, the prompt to complete Strokehealth digitally caused stress for participants with less experience using technology. Additionally, the web-based system of the patient portal was perceived as difficult to navigate, adding to the digital stress. Some participants also experienced stress related to clicking the prompt to open Strokehealth and expressed concern about clicking the wrong button. However, once participants successfully managed to navigate the patient portal, most considered it easy to complete Strokehealth. For participants with severe technological issues, it was considered advantageous to have someone assist them. Additionally, some participants supported the idea of being able to choose between the digital previsit tool and a paper version on which they could make notes with a pen and then refer to during the follow-up visit.

##### Clarity and Purpose in the Introductory Text of Strokehealth Supports Usability

The participants’ perception of the initial introductory text had an impact on their willingness to fill out Strokehealth. Most participants appreciated that the text was short and clear. Based on the contents of the introductory text, all participants clearly understood the purpose of answering the questions before their follow-up visit. Participants appreciated that the introductory text explicitly stated that there were only 14 questions, estimated to take a reasonable amount of time. Specific information that participants found helpful included that the answering process could be initiated at any preferred time and place and that the upcoming follow-up visit would provide an opportunity to elaborate on any of the indicated symptoms (ie, “yes” responses in Strokehealth).

Participant 25: Well, if it [the introductory text] says that your nurse will ask you about answers, so that you can explain how you have thought, then it makes you answer [the question]. Well, then I answer “yes” when it comes to swallowing because then I know that I can explain that later. And pain, I have that, but I can explain how much pain it is. [Female, 73 years old]

However, participants suggested providing the information that one could take a break at any point and making it clear that they could revisit and review their answers before submitting Strokehealth. On the other hand, participants expressed concerns that adding too much new information to the introductory text could impact readability.

Despite previous adjustments extending the introductory text, several participants did not notice the link to the advisory text at the end of the tool (or forgot it while answering the questions), suggesting a need for further improvements in future versions. Others acknowledged seeing the link to the advisory text but opted not to read it, either because they no longer had symptoms or because they believed that they had already received sufficient information.

#### The Wording and Structure of Strokehealth Influences the Response Process

This theme highlights how the linguistic formulation and structural design of Strokehealth affect users’ understanding, interpretation, and response behavior. The response process was also perceived as dependent on individual prerequisites, highlighting the importance of how information is presented, the clarity of instructions, and the perceived user-friendliness of the interface.

##### Explanatory Texts Shape Interpretations of Questions

Most participants saw no disadvantages to the explanatory texts, and some even found them crucial for interpreting the questions and understanding their meaning. The provided contextual information—including various examples related to specific health issues—prompted participants to make new associations related to their own impairments. These reflections helped participants provide more accurate and informed answers to the questions. On the other hand, one participant expressed dissatisfaction with the comprehensive list of possible ailments in the explanatory text. He was concerned that selecting “yes” would inaccurately suggest that he experienced all listed ailments. Another participant noted that although some questions, for example, “Do you have more difficulty walking or moving safely after a stroke?” could include minor symptoms, such as slightly impaired balance, these symptoms were not mentioned within the explanatory text. He therefore found this question difficult to respond to.

Participants expressed overall satisfaction with the explanatory texts, finding them easy to comprehend. The texts were deemed concise and straightforward, and not disruptive when answering the questions. Participants emphasized the importance of maintaining simplicity and noted that excessive length could reduce response rates. However, participants identified certain problem areas that could benefit from more detailed explanations. For instance, participants found the question about pain difficult due to the complexity of the condition.

Participant 4: Perhaps the definition of pain is a bit subjective too. I don’t have severe pain; it’s more like a pressure sensation. But of course, that could also be considered pain, depending on how one defines pain—whether it’s sharp pain, severe pain, discomfort, or something like that. I’m not sure if I would have answered ‘pain’ even though I had this slight discomfort. It didn’t hurt; it’s more of a discomfort if anything, especially if it’s a milder sensation that doesn’t feel like direct pain. [Male, 58 years old]

Not everyone felt a need to read the explanatory texts. Some participants chose to read the attached text only if the question sparked their interest in the topic, was unclear, or prompted deeper reflection on their remaining symptoms.

Participant 22: In those cases, my answer was obvious, I didn’t read all of them thoroughly. I didn’t go into the details or the fine print—I just moved on to the next one. I didn’t read everything all the time; if the question was crystal clear to me, I just went to the next one without any comments. Yes, there were certain questions—not all of them—but some where I thought, “What the hell do they mean by that?” And of course, in those cases, I had to read more carefully to understand what it actually said. [Male, 65 years old]

##### Individual Prerequisites Influence the Response Process

During the interview, several participants reported experiencing communication or memory problems, including signs of aphasia. For many, these impairments impacted their ability to recall details, understand, and express their thoughts effectively within Strokehealth. Some participants mentioned that having a supportive spouse was advantageous when completing Strokehealth. Others mentioned that they answered the questions quickly, either due to their natural tendency to complete questionnaires quickly or because they were at work when the notification arrived. Responding rapidly may have reduced the time available for reflection on the questions; however, participants did not spontaneously raise this concern during the interviews.

Participant 6: It could be that I took a break from work when I did it, and maybe I felt like I didn’t really have time to continue or look into it further, so I felt done. That’s often how it feels—when I’ve completed a survey, I just want to get on with my life [laughs]. [Male, 46 years old]

Participants commonly mentioned the problem of fluctuating mental fatigue since their stroke. When participants experienced mental fatigue during their completion of Strokehealth, it influenced every answer. Participants felt that it was misleading to answer “yes” to questions about difficulties with activities of daily living if they were typically able to manage but were currently hindered by fatigue. They suggested including a specific question about fluctuating mental fatigue or adding a free-text area where participants could indicate “sometimes” instead of being limited to the binary “yes” or “no” responses. Another suggestion was to move the question about “life after stroke” from the bottom to the top of the tool, since that question was related to fatigue and hence impacted their responses to the subsequent questions. Notably, the presence of mental fatigue significantly affected participants’ ability to focus during the response process. One participant described feeling mentally sluggish, as if their thoughts were moving “as slow as toffee.” Accordingly, participants highlighted the need for flexibility to take breaks, without fear of losing previous responses.

##### Importance of Optimizing User-Friendliness and Allowing for Simplicity

Participants provided valuable insights regarding how to improve the tool’s design to enhance the response process. Most participants found the structure, layout, and language of Strokehealth to be attractive, educational, and easy to comprehend. The number of questions, response options, wording, and sequencing of questions were all generally deemed user-friendly and straightforward. No language was considered offensive. In particular, participants with mild poststroke symptoms found the tool easy to navigate.

Regarding the response options, participants speculated that binary choices (“yes” or “no”) might be insufficient, suggesting a need for more nuanced options.

Participant 6: I mean... I answered “yes” for those where I had even the slightest symptom. I did, yes. That’s how I interpreted it: if I experienced even the slightest symptom, I would answer “yes,” and if I didn’t, I would answer “no.”

Interviewer: So it wasn’t hard for you to understand that this is how you should answer, at least?

Participant: No, no. But the feeling could still be that this “yes” might not convey the full truth. [Male, 46 years old]

Additionally, they emphasized the importance of providing a free-text area below each question to allow for personalized responses and improve user-friendliness. Participants felt that a single free-text area at the end of the tool was insufficient because they often wanted to specify directly after each question. The need for a free-text area was particularly evident after the question “Life after stroke,” which was generally considered quite broad, with the ambition of capturing many aspects of importance.

Participant 2: I just think I would have liked to answer “yes,” but then define it. I would want to specify right under that question... I would like to specify them a bit more, either by breaking that question into several or by allowing a free-text option right after, where you can specify what you mean. [Male, 61 years old]

Not all participants shared this perspective. Some found that the combination of the question itself and a single free-text area at the end was sufficient—especially if they found it challenging to write due to linguistic or motor impairments. Participants also reflected on the answer option “choose not to answer,” which some viewed as somewhat peculiar, while others saw it as a “way out.” No specific suggestions were made for alternative wording to enhance clarity, but some reflected that this response option could be supplemented with a free-text area to allow individuals to explain why they chose not to answer.

Participants appreciated that a summary appeared when they completed the response process before submitting Strokehealth. Some suggested that the “submit” button should be made more prominent and visible. After submitting Strokehealth, it disappeared from the screen, which was seen as a disadvantage if someone wanted to review their answers before their follow-up visit. Similarly, it was suggested that it should be made easier to print the summary of their responses.

### Development and Amendments to Version 2.0

Strokehealth was amended and altered based on the findings from the individual interviews and the theoretical framework—for example, aspects of person-centered care and health literacy (Multimedia Appendix 4). Several important changes were made in version 2.0, including a revision of the introductory text to better align with expressed preferences. Additionally, the answer options were extended to include a free-text box below each question and additional space in the general free-text box at the end of the tool. Moreover, the explanatory and advisory texts were improved to exhibit greater consistency in structure and content. These included confirming experiences, suggestions of what one can do by oneself (self-management), suggestions of potential interventions, and a prompt to seek support, if needed. Notably, the platform for the national portal has some limitations regarding layout options, predefined typography, and colors that cannot be changed.

## Discussion

### Principal Findings and Comparison With Prior Work

#### User Experience and Perceived Usefulness

The Strokehealth digital previsit tool, when used before a scheduled follow-up visit after stroke, was perceived as a supportive aid for facilitating dialogue and identifying patient needs. It was also considered to be useful and user-friendly. Themes identified from the interviews emphasized the importance of patient comprehension and motivation to initiate the response process. The real-world experiences captured in this qualitative study, combined with the findings from the initial patient satisfaction survey (part 1 [17]), provided the foundation for further improvements to the tool. While the initial survey provided useful data on general user satisfaction and perceived usability, the subsequent qualitative evaluation offered deeper insight into patients’ experiences and contextual factors affecting their interaction with the tool. It captured emotional responses such as feeling acknowledged and supported, practical barriers related to digital literacy, and the need for more nuanced response options. These findings informed concrete improvements to the tool and highlighted the importance of considering patients’ individual circumstances when implementing digital previsit tools in routine care. Based on user feedback, minor adjustments were recommended in version 2.0 to better support the follow-up process from initiation to identifying patient needs. Additionally, participants suggested greater flexibility in the timing and method of introducing Strokehealth to patients to enhance its accessibility and usability.

#### Supporting Patient Engagement and Self-Management

The current findings indicate that the information presented in Strokehealth fosters increased patient engagement and potentially supports meaningful dialogue with both health care professionals and family members. The stroke-specific information addresses common knowledge gaps that are often experienced by people with stroke and their families [4,23], especially after first-time strokes [24]. Participants reported that the information reassured them that their experiences were normal and, in some cases, motivated them to seek additional resources on the web or take health-related actions. Patients require relevant, concise, and easily accessible information to be able to make informed decisions and support their self-management [4,23,25]. The concise information within Strokehealth aims to foster shared responsibility for health—aligning with the broader health care shift toward self-management [26]; however, simply providing information is insufficient to elicit meaningful behavior changes without active patient engagement [25] or effective communication with health care providers [4]. Patient health engagement can be further encouraged by combining Strokehealth with a follow-up visit (whether on-demand or in-person) during which patients can revisit information and discuss it with health care professionals.

#### Self-Reflection and Response Design

Participants in this study emphasized the importance of self-reflection during the response process, highlighting various aspects of its significance. First, the response process encouraged participants to consider issues they might not have otherwise reflected upon. Somewhat surprisingly, participants also expressed that the response process fostered a sense of gratitude about not being more severely affected, as well as feeling cared for by the health care system, rather than abandoned—a sentiment that aligns with previous research findings [6]. Second, the self-reflections and responses were influenced by how participants interpreted the explanatory texts attached to each question. Most participants found these texts to be sufficient; however, some expressed that they did not fully address subtle impairments. Third, self-reflection was influenced by the design of the response options. Participants expressed their desire to provide more nuanced responses than were allowed by the binary response options, which reinforced findings from previous evaluations of the co-design process [14]. While this limitation was acknowledged, participants with firsthand experience of a stroke indicated that the response options were generally effective when complemented by a free-text area with sufficient space to record their reflections. Following the evaluation, restructuring the questions to include additional response options aimed at capturing milder symptoms was considered. However, overly complex or ambiguous questions could hinder engagement. Therefore, this approach was ultimately deemed to introduce unnecessary complexity, potentially compromising the goal of developing a user-friendly tool. Based on these insights, Strokehealth version 2.0 was modified to incorporate free-text areas below each question and revised text to better capture the nuances of patients’ experiences and support the response process.

#### Accessibility and Digital Literacy

The present results suggest that Strokehealth can be accessible to a broad range of patients with stroke if choices are offered regarding when and how the tool is introduced. Some participants described experiences of “digital stress” when navigating the patient portal, although 48 out of 58 participants (82%) used the web on a daily basis. This highlights the need for Strokehealth to be accessible to individuals with limited experience using digital tools. It is recommended that health care providers consider patients’ eHealth literacy levels [27] and implement alternative communication methods as needed. For patients with limited digital literacy—including low digital skills, low confidence, or reluctance to use digital tools—health care providers could encourage seeking support from friends or family to alleviate digital anxiety. Our results indicate that some participants successfully completed the tool with support from spouses. Additionally, alternatives to the digital version should be readily available to help build patients’ confidence in their ability to self-manage. Therefore, Strokehealth is also offered as a paper version [28], which can be sent by a health care professional to a patient who chooses not to interact with the patient portal after receiving the initial text message. Such efforts can improve accessibility and organizational health literacy, ultimately supporting patients in more effectively navigating the health care system [29].

#### Implementation Considerations

Importantly, evaluations of a digital health tool such as Strokehealth must consider not only the functionality of the tool itself but also the contextual factors [16] related to the follow-up process. Effective introduction is crucial for engaging patients in the proactive management of their health [30], including clear communication of the purpose of a notification and building trust in the sender [31]. In this study, the timing and method of delivery played a key role in engagement with the tool, with some participants initially questioning the legitimacy of the digital notification. Mentioning Strokehealth upon hospital discharge could raise awareness about the tool, enhance trust, and help patients perceive the follow-up process as seamless and integrated. Ideally, patients could be informed at the time of hospital discharge that they will later receive a digital previsit tool, once they have spent some time at home. This information should include the purpose of Strokehealth, and if they choose to opt out, a paper version could be offered at that later stage. Engagement was further influenced by factors such as fatigue and cognitive impairment, underscoring the need for well-timed notifications, ideally in the morning when patients’ energy levels are higher. Strokehealth version 2.0 addresses these challenges by promoting flexibility, allowing patients to complete the tool at their convenience and to take control of their follow-up process.

#### Implications for Digitally Supported, Person-Centered Care

Our findings align with previous calls for digitally supported, person-centered care in long-term conditions such as stroke [1,2]. By providing insights into how patients experience the use of a digital previsit tool, this study contributes to the growing evidence base on how eHealth interventions can promote reflection, engagement, and preparedness ahead of follow-up visits.

### Strengths and Limitations

This study involved patients who were consecutively recruited by the selected health care professionals; therefore, bias due to selection cannot be excluded. However, this sample—the majority of whom had a mild stroke—is representative of the Swedish population. The comprehensive description of the context and participants in this study, complemented by part 1 [17], supports the transferability of the findings to other organizations. In accordance with the co-design method, the researchers were involved in all parts of the design process [32,33], including the evaluation elements. However, the researchers’ involvement could have made participants feel hesitant to express drawbacks, despite encouragement to openly share thoughts. Some of the interviews were relatively short in duration, which may have limited the depth of participants’ responses. It is possible that conducting the interviews face-to-face, rather than by telephone, could have facilitated richer and more nuanced accounts by allowing for nonverbal communication and a more personal connection between the interviewer and the participant. Nevertheless, the critical feedback and rich data in this study demonstrate participants’ willingness to share their experiences and contribute to improving the tool. To enhance reflexivity, reflections were systematically noted. Importantly, the researchers’ preunderstanding is considered a factor that influences the data analysis and interpretation [34]. To ensure confirmability, the impact of this preunderstanding was considered throughout the analysis, together with continuous return to the coded data and transcripts in NVivo. Moreover, credibility was strengthened by conducting the interviews in a familiar environment, using a telephone interview. However, the use of the telephone could have influenced participant recruitment. Interviews were conducted by telephone due to the COVID-19 pandemic; however, this was found to be a surprisingly well-functioning method of collecting data in this sample.

The Strokehealth digital tool has now been tested within a clinical care pathway to assess whether patient needs and preferences are being appropriately addressed in accordance with prior recommendations [16]. Accordingly, Strokehealth has been implemented within the Swedish health care system, allowing health care professionals to access patient responses via the national patient portal prior to clinical visits. However, the broader implications and potential benefits from a health care system perspective warrant further exploration. Following guidelines for evaluating complex interventions, the next step would be to gather the perspectives of health care professionals to understand how Strokehealth could support them in the delivery of care. Together, our studies (parts 1 and 2) indicate that the tool’s overarching purpose—to encourage reflection and preparation for a planned health care visit—remains broadly applicable among patients with stroke.

### Conclusions

The digital previsit tool Strokehealth was generally well received, with participants describing it as supportive, easy to use, and valuable for fostering reflection and preparing for meaningful dialogue. Patients’ feedback led to concrete refinements in Strokehealth version 2.0, including improved explanatory texts, added flexibility in the timing and format of delivery, and expanded opportunities for free-text input. Importantly, the findings highlight that digital tools must account for individual circumstances, including eHealth literacy and cognitive or emotional challenges, in order to ensure equitable access. This study contributes to the evidence base for implementing person-centered, digitally supported follow-up care in stroke rehabilitation.

## Appendix

Multimedia Appendix 1 Strokehealths' 14 questions.

Multimedia Appendix 2 COREQ checklist.

Multimedia Appendix 3 Semistructured interview guide.

Multimedia Appendix 4 List of amendments to Strokehealth version 2.0.

## Abbreviations

COREQ: Consolidated Criteria for Reporting Qualitative Research
