# Supplementary material for: Patients’ Perceptions of Using a Digital Previsit Tool in Outpatient Settings (Part 2): Qualitative Study
Source: JMIR Hum Factors. 2025 Oct 6;12:e73477. doi: 10.2196/73477 (PMC12538183; doi:10.2196/73477)
Supplement: Multimedia Appendix 1 [file humanfactors_v12i1e73477_app1.docx]

## Multimedia Appendix 1: Strokehealths’s 14 questions

**(Introductory text)**

How have you been affected by your stroke?

Before your appointment, you will be asked 14 multiple-choice questions about common health problems seen after a stroke. You can answer Yes regardless of whether the impact is large or small. Answering the questions will give you an opportunity to think about what is important for you to discuss during the appointment. If you want to add anything, there is a comment box at the end of the questionnaire.

Remember that you will also have the opportunity to expand on your answers when speaking with your healthcare provider. Together, you can decide what care and rehabilitation is right for you. Even if several areas of your life have changed, you usually have the opportunity to influence your own health.

You can answer the questions now, or at a time that suits you better.

Brief information:

Follow the link below to read and think about which points concern you. You can also read about what support is available from different healthcare professionals.

(clickable link to the advisory text)

**Preventing another stroke**

**1. Do you want advice on what you can do to prevent another stroke?** Max. 1 answer

Yes#/No#/Choose not to answer#

Comments (optional):

Many people worry about having another stroke. The risk of having another stroke can be significantly reduced through a combination of healthy lifestyle, medication, and blood pressure checks.

**Activities of daily living**

**2. Do you find it more difficult to take care of yourself after your stroke?**

Yes#/No#/Choose not to answer#

Some people find it difficult to handle activities of daily living, such as showering, dressing, preparing meals, or leaving the house.

**Do you have difficulty dressing, washing, or showering on your own?**

Yes#/No#/Choose not to answer#

**Do you have difficulty preparing hot drinks or meals?**

Yes#/No#/Choose not to answer#

**Do you have difficulty leaving the house?**

Yes#/No#/Choose not to answer#

**Walking and mobility**

**3. Do you find it more difficult to walk or move about safely after your stroke?**

Yes#/No#/Choose not to answer#

Even slight balance problems can increase your risk of falling. You may have more difficulty moving around indoors, for example moving from your bed to a chair, or navigating your way around the bathroom. In a more demanding outdoor environment, some people find it difficult to ride a bus or walk on uneven surfaces, et cetera.

**Muscle tension and stiffness (Spasticity)**

**4. Have you experienced an increased stiffness in your arms, hands and/or legs after your stroke?**

Yes#/No#/Choose not to answer#

Some people experience increased muscle tension and stiffness. You may have noticed difficulty in controlling your movements. Treatment can prevent pain or stiffness, and improve your chances of rehabilitation.

**Does the stiffness interfere with your activities of daily living?**

Yes#/No#/Choose not to answer#

Many people find that the increased tension in their muscles can make it more difficult to get dressed and manage their hygiene, et cetera.

**Pain**

**5. Have you experienced any new pain after your stroke?**

Yes#/No#/Choose not to answer#

Some people experience pain after a stroke. You may have pain in your muscles and joints, or in your head. There are different types of pain, such as nerve pain.

**Bladder or bowel control**

**6. Do you have more of a struggle to control your bladder or bowels after your stroke?**

Yes#/No#/Choose not to answer#

Some people experience problems with involuntary leakage, incomplete emptying of their bladder, or difficulty getting to the toilet in time. You may have noticed problems with constipation or diarrhoea.

**Communication**

**7. Do you find it more difficult to communicate with others after your stroke?**

Yes#/No#/Choose not to answer#

Not being able to speak or make yourself understood is often perceived as a barrier in the activities of daily living. You may find it difficult to express yourself or understand what others are saying. Some people find it difficult to pronounce words clearly, while others have difficulty reading and writing.

**Mood/Mental health**

**8. Do you feel more anxious or depressed after your stroke?**

Yes#/No#/Choose not to answer#

Some people feel dejected or anxious, become more sensitive to stress, and experience mood swings after a stroke. More severe conditions can lead to depression, which is a medical condition that should be treated.

**Cognition**

**9. Do you have more difficulty thinking, concentrating, or remembering after your stroke?**

Yes#/No#/Choose not to answer#

Some people experience difficulties with getting started, doing more than one thing at a time, and solving various problems. You may have noticed that your abilities vary in different situations. Activities that can be challenging include reading, paying bills, and remembering scheduled appointments.

**Follow-up question for Cognition**

**Does this interfere with your participation in activities?**

Yes#/No#/Choose not to answer#

Some people find that they perform mental tasks differently depending on their circum­stances. You may need to rest or take more breaks to be able to stay active for a full day.

**Life after stroke**

**10. Do you find it more difficult to do things that are important to you after your stroke?**

Yes#/No#/Choose not to answer#

Many people experience difficulties in areas such as leisure, work, sex and intimacy, and socialising with friends and family. You may feel uncertain about returning to activities you did before your stroke. Some experience an increase in fatigue. It can be easy to withdraw and not take part in the same activities as before.

**Relationship with family**

**11. Has your relationship with your family become more complicated or stressful after your stroke?**

Yes#/No#/Choose not to answer#

Life often changes after a stroke – both for you and your loved ones.

**Oral health**

**12. Do you find it more difficult to manage your oral hygiene after your stroke?**

Some people find it more difficult to brush their teeth or dentures. Poorer dental health after a stroke may be due to a number of reasons that need to be investigated.

Yes#/No#/Choose not to answer#

**Eating and swallowing**

**13. Do you find it more difficult to eat or swallow after your stroke?**

Some people find it more difficult to chew, and choke more easily after their stroke. You may cough more than before when you eat or drink. Some people have trouble getting enough nutrition.

Yes#/No#/Choose not to answer#

**Other challenges**

**14.** **Do you have any other concerns after your stroke that affect your recovery or cause you difficulties?**

Yes#/No#/Choose not to answer#

The consequences after a stroke can take different forms and may even vary over time. Not everything is covered in this questionnaire. You can raise any questions or concerns you have during the consultation.

**Closing questions:**

**Is there anything else you would like to say before your healthcare visit? (free text)**

**Do you have any rehabilitation interventions related to your stroke right now?**

Yes#/No#/Choose not to answer#

This refers to whether you receive help to adapt your training to your needs, with the support of rehabilitation professionals. This can be individual, group or self-training programmes.

**Who completed this questionnaire?** Max. 1 answer

I___I = Me, unassisted

I___I = Me, with the help of a relative or healthcare professional

I___I = Relative only

I___I = Other

**With the right support, you can influence your health**

At your healthcare visit, you and your healthcare provider can discuss what you can do on your own, as well as what support you can get from different healthcare professionals.

Your healthcare provider has general knowledge about strokes, but you are an expert on your specific health problems and situation. Together with your healthcare provider, relatives, and other key support people, you can be involved and share responsibility for your health.

If you have a care plan from a previous healthcare provider, you can bring it along to the visit. If you share your thoughts, the healthcare provider can help to tailor your care to your needs.

Once you have **confirmed your answers**, you will be taken to a final page with a link. By reading the brief information, you can think about what is important for you to bring up at the visit.

“Confirm your answers” –

(NEW PAGE)

Important information to read before the follow-up appointment

Clickable link to the advisory text.

NEW PAGE (Advisory text, received when following the link)

With the right support, you can influence your health

By reading this information, you can prepare for your healthcare visit. Think about which points concern you.

At your healthcare visit, you and your healthcare provider can discuss what you can do on your own, as well as what support you can get from healthcare professionals. Your healthcare provider has general knowledge about strokes, but you are an expert on your specific health problems and situation.

Remember that improvements can take place over a long period of time after a stroke.

**1. Preventing another stroke**

The risk of having another stroke can be significantly reduced through a healthy lifestyle, medication, and blood pressure checks. You can make a difference by being physically active, eating a healthy diet, not smoking, and reducing your alcohol consumption.

Changing lifestyle habits can take time, and you may need help to do this. Talk to your doctor or nurse about your needs.

**2. Activities of daily living**

It can be more difficult to cope with everyday life after a stroke. For most people, resuming activities that they used to do helps their recovery.

Sometimes you may need to do activities in a new way in order to do what is important to you. You can get support from an occupational therapist, who can give you advice, training, and aids to help you cope with everyday life.

**3. Walking and mobility**

Some people find it more difficult to walk and move around after a stroke. Improvements can take place over a long period of time. You can do exercises to improve your walking and balance, so you can move around more easily and reduce your risk of falling. Aids can also help.

Think about your own goals and how you can be physically active in a way that suits you. You can get support from a physiotherapist or the rehab team.

**4. Muscle tension and stiffness (Spasticity)**

For some people, increased muscle tension can lead to pain and stiffness after a stroke. If you are experiencing problems, advice and treatment can help your rehabilitation.

You can get personalised support from a healthcare professional, such as a physiotherapist or occu­pa­tional therapist. Your doctor can help you with further investigation and suggest appropriate measures.

**5. Pain**

Some people experience pain after a stroke. If you are in pain, you may need help to investigate the cause in order to get the right treatment.

A physiotherapist or doctor can investigate and suggest appropriate measures. You can get support from a physiotherapist if the pain is caused by muscle or joint problems.

**6. Bladder or bowel control**

Some people find it difficult to hold their bladder and make it to the toilet in time after a stroke. You may have noticed problems with constipation or diarrhoea. If you are having problems, contact your health centre.

You can get support and information from a nurse who is knowledgeable about incontinence problems after a stroke. There are good aids available to make everyday life easier.

**7. Communication**

Some people find it more difficult to communicate after a stroke. Speech often improves over time, and most people learn to cope with this in their everyday life. One way to manage this is to speak to fewer people at a time, or talk at a slower pace.

If you have difficulties, a speech therapist can assess, treat, support adaptations, and try out communi­ca­tion aids. If you would like more information and support, you can also contact Afasiförbundet [Aphasia Association].

**8. Mood/Mental health**

Some people find that their mental well-being is affected after a stroke. If you are struggling, daily routines, taking walks, and telling someone how you are feeling can help you feel better.

If you are experiencing major problems, do not wait to seek help. Talk to your doctor, nurse, or psychologist. The problems may be eased through medical treatment or counselling. If the problem feels urgent, contact sjukvårdsupplysningen [the healthcare guide] on 1177, or call 112.

**9. Cognition**

For many people, problems with for example memory and concentration become noticeable when they return to their daily routines. If you notice problems, you can often find new ways to do activities.

A calm environment and doing just one thing at a time can help. You can also use assistive devices to better manage your daily life. You can bring this up with your rehab team or your doctor, who can refer you further.

**10. Life after stroke**

You are the best judge of how you need to live to feel good. Some people find new interests after their stroke. It is a good idea to join a support group or patient association, such as Strokeförbundet [Swedish Stroke Association], Afasiförbundet [Aphasia Association] or Neuroförbundet [Neuro Association]. It may help to know that you are not alone.

If you are planning to return to work, a rehab coordinator or your doctor can help you with planning and relevant contacts. If you are experiencing severe fatigue, you may need an individual assessment to find out the cause. The rehab team can help you find ways to make your daily life easier.

**11. Relationship with family**

For many people, having a stroke leads to a change in their lives. There are family support workers in all municipalities who can be a good source of support. By joining a support group, you and your family can meet others in a similar situation and see that you are not alone.

Patient associations can be a good source of support, for example Strokeförbundet, Afasiförbundet or Neuroförbundet. Sometimes relatives can come with you to a healthcare visit to get information and answers to their questions.

**12. Oral health**

The risk of tooth decay may increase after a stroke. You can prevent tooth decay by maintaining good oral hygiene. It is a good idea to make an appointment with your dentist a few months after a stroke. If you have oral health problems, you can apply for special dental care assistance.

**13. Eating and swallowing**

It is important to get enough nutrition to recover properly. You can get good advice or a referral from your nurse.

If you have difficulty swallowing, a speech therapist can make an assessment and give you advice. Swallowing can be made safer through assistive technology and adaptations.

**14. Other challenges**

People are affected in different ways by their stroke. The problems are often hidden from others. Raising your questions or concerns can make it easier for you to get the support you need.

With the right support, you can influence your health

Together with your healthcare provider, relatives, and other key support people, you can be involved and share responsibility for your health.

If you have a care plan from a previous healthcare provider, you can bring it along to the visit. If you share your thoughts, the healthcare provider can help to tailor your care to your needs.

The Strokehälsa [Strokehealth] has been developed within the university of Gothenburg in collaboration with Strokeförbundet the stroke association in line with the Swedish national guidelines for stroke.
