# Supplementary material for: Patients’ Perceptions of Using a Digital Previsit Tool in Outpatient Settings (Part 2): Qualitative Study
Source: JMIR Hum Factors. 2025 Oct 6;12:e73477. doi: 10.2196/73477 (PMC12538183; doi:10.2196/73477)
Supplement: Multimedia Appendix 3 [file humanfactors_v12i1e73477_app3.docx]

## Multimedia Appendix 3

Semi-structured interview guide

**Introduction**

- Introduction with background of the study, aims, and structure of the interview
- Check for provision of informed consent and permission for audio-taping

**Opening questions**

- You have now had the opportunity to use Strokehealth and likely have many thoughts about how the questionnaire works. When you think about using Strokehealth after a stroke, what are your immediate thoughts?

**Key questions**

- What was your initial reaction when you received the message from the 1177* about completing the Strokehealth questionnaire?
- How did you experience answering the questions in Strokehealth?
- How did you perceive the information provided in Strokehealth?
- Now that you are having a follow-up appointment, what significance does it have for you that you completed this beforehand?

**Closing questions: areas for improvement**

- If you could change something in Strokehealth, what would you change?
- What advice would you give us for the continued development of Strokehealth?

**Closing**

- Is there anything else you would like to add?
- Thank you statement and closing.

* 1177 refers to Sweden’s national healthcare information and advisory service which serves as the access point for the national patient portal, where citizens can communicate with healthcare providers and access digital health services.
