# Supplementary material for: Patients’ Perceptions of Using a Digital Previsit Tool in Outpatient Settings (Part 2): Qualitative Study
Source: JMIR Hum Factors. 2025 Oct 6;12:e73477. doi: 10.2196/73477 (PMC12538183; doi:10.2196/73477)
Supplement: Multimedia Appendix 4 [file humanfactors_v12i1e73477_app4.docx]

| **Version 1.0:**  Tested with patients before a follow-up visit. | **Version 2.0:**  Suggested amendments based on findings in current study, |
| --- | --- |
| Integrated in the regulated health platform 1177 demanding secure log in. |  |
|  | The logo with green leaves was added at the end, before submitting the answers. |
|  |  |
| Q1“*Do you want* information about how you can prevent another stroke” |  |
|  | #3Heading changed to ”Walking and mobility”. |
|  | PSC, Q4 Heading: ”Muscle tension and stiffness (Spasticity)” |
|  | PSC Q5 “Have you experienced any new pain after your stroke?” |
|  | PSC Q6 Heading: “Bladder or bowel control” |
|  | PSC Q8 Heading: ”Mood/Mental health” |
|  | PSC Q10 “Do you find it more difficult to do things that are important to you after your stroke?” |
| “After your stroke” was moved to the end of each question |  |
|  |  |
| Additional alternative, “choose not to answer” |  |
| Additional alternative, ”choose not to answer” |  |
| Additional areas were added: Eat and drink and oral health. | Minor amendments |
|  | A free-text box gets available underneath each question, after they have chosen their response option. |
|  |  |
| Minor amendments. | Information about the link was moved.  Amendments including answering process, availability of a comment box at the end and a prompt to choose an optimal time. |
|  |  |
|  |  |
| Further amendments of wording and balancing general descriptions with specific examples e.g., fatigue mentioned in Q10. | Amendments of wording and structure for consistency and clarity. Refinements to better confirm difficulties at different levels. |
|  |  |
| Heading “Do on your own and get support” | Heading: “With the right support, you can influence your health” |
|  | The link is placed in a “box” before the questions and after submit.  A short information about its purpose.  Amendments of wording for clarity. Revised structure of the content for consistency exemplified as follows: 1) some people experience… 2)if you notice problems.. 3) it can get better if you…4) the rehabilitation team can provide support… |
